# ADAM10-cleaved ephrin-A5 contributes to prostate cancer metastasis

**DOI:** 10.1038/s41419-022-04893-8

**Published:** 2022-05-12

**Authors:** Chenchen Cai, Miaomiao Zhang, Lei Liu, Haoliang Zhang, Yi Guo, Ting Lan, Yinhai Xu, Ping Ma, Shibao Li

**Affiliations:** 1 Medical Technology School of Xuzhou Medical University, Xuzhou, 221004 China; 2 Xuzhou Central Hospital, The Affiliated Xuzhou Hospital of Medical College of Southeast University, Xuzhou, 221009 China; 3 Department of Laboratory Medicine, Affiliated Hospital of Xuzhou Medical University, Xuzhou, 221002 PR China; 4 Department of Physiology, Xuzhou Medical University, Xuzhou, 221004 PR China

**Keywords:** Mechanisms of disease, Diagnostic markers

## Abstract

A disintegrin and metalloprotease-10(ADAM10) promotes the metastasis of prostate cancer (PCa), but the specific mechanism is indistinct. Herein, DU145 cell lines with stable overexpression and knockdown of ADAM10 were constructed. We found that ectopic expression of ADAM10 not only significantly facilitated cell proliferation, migration, invasion, and inhibited apoptosis, but also could specifically hydrolyze ephrin-A5 and release the ephrin-A5 soluble ectodomain into extracellular media in vitro. These effects were reversed by ADAM10 depletion or treatment of GI254023X. Meanwhile, the co-location and physical interaction among EphA3, ephrin-A5, and ADAM10 were observed in PCa cells using immunofluorescence and immunoprecipitation techniques. Interestingly, overexpression of EphA3 exerted opposite effects in DU145 (ephrin-A5 + ) cells and PC-3 (ephrin-A5 ± ) cells. In addition, the pro-tumor function of EphA3 was reversed by the treatment with the exogenous ephrin-A5-Fc, which increased the phosphorylation level of EphA3 in PC-3 (ephrin-A5 ± ) cells. In nude mice, ADAM10 accelerated growth of the primary tumor, decreased the level of ephrin-A5 in the tumor tissue, but increased the level of ephrin-A5 in the peripheral blood, accompanied with an increase in the expression of CD31 and VEGF (vascular endothelial growth factor) in the tissue. What is more, the serum ephrin-A5 content of patients with metastatic PCa was significantly higher than that of the non-metastatic group (*P* < 0.05). The receiver operating characteristic curve(ROC) showed that the area under the curve(AUC) of serum ephrin-A5 as a marker of PCa metastasis was 0.843, with a sensitivity of 93.5% and a specificity of 75%. It is concluded that ADAM10-mediated ephrin-A5 shedding promotes PCa metastasis via transforming the role of EphA3 from ligand-dependent tumor suppressor to ligand-independent promoter, and ephrin-A5 in the blood can be used as a new biomarker for PCa metastasis.

## Introduction

Prostate cancer (PCa), the most commonly diagnosed non-cutaneous cancer in men of western world, is the second leading cause of cancer-related death [1]. Many young people have initial histological changes many years before clinical symptoms appear, suggesting PCa tends to develop at younger ages [2]. Patients with metastatic PCa have a higher risk of death and short survival [3], which makes metastasis remain the main cause of death from PCa [4, 5]. It is of great social significance to explore the mechanism of PCa metastasis and search for relevant diagnostic markers or therapeutic targets. ADAM10, an important shedding enzyme for membrane-anchored proteins such as growth factors, receptors and adhesion proteins and so on, is widely expressed in various tissues and cells [6, 7]. Since ADAM10 plays essential roles in cell adhesion, cell migration, protein lysis, intracellular signal transduction, and the generation and development of various tumors in vivo [8–11]_,_ it has become a hotspot in the field of tumorigenesis and therapy study these years [12]. ADAM10 is highly expressed in a variety of solid tumors, including PCa, and promotes tumor deterioration [13]. ADAM10 participates in the malignant transformation of prostatic epithelial cells by hydrolyzing amphiregulin (AREG), the ligand of epidermal growth factor receptor (EGFR), which is a member of the receptor tyrosine kinase family [14]. However, the AREG hydrolysis of EGFR ligand mediated by ADAM10 does not affect the malignant transformation of PC-3 cells [15], suggesting that ADAM10 may participate in PCa metastasis through other mechanisms than the hydrolysis of EGFR ligand. These mechanisms of ADAM10 need to be further studied.

EphA3, an essential regulatory target of ADAM10 through cleaving its primary ligand ephrin-A5, is a member of the receptor tyrosine kinase family, and has been reported to have dual biological effects in tumor progression [16]. Up-regulation of EphA3 in PCa is associated with accelerated cell migration and invasion, increased angiogenesis, and a poor prognosis [17]. Conversely, EphA3 suppressed tumors by disrupting the tumor stromal microenvironment in mouse xenografts [18]. These opposing roles may be regulated by its primary ligand ephrin-A5, which induces the phosphorylation of EphA3 receptor and triggers the downstream signal transduction involved in tumor suppression [16]. EphA3 and ephrin-A5 exert biological effects through the formation of complexes bound by receptors and ligands [19–23], and ephrin-A5 is the most suitable ligand for EphA3 [24]. The absence of ephrin-A5 activates EphA3 signaling via ligand-independent pathways that promotes tumor progression [25, 26]. What is more, serum proteomic analysis has confirmed that serum ephrin-A5 is a preferable prognostic marker for patients with castration-resistant prostate cancer (CRPC) [27]. Paradoxically, our previous study on ephrin-A5 expression in PCa tissues showed that 65% (13/20) of PCa tissues were weakly positive or even negative [28], which is consistent with the expression of ephrin-A5 in other tumor tissues [29]. The above contradictory findings prompted us to speculate that the shedding of ephrin-A5 by ADAM10 may result in the differential expression of ephrin-A5 between PCa tissue and patient's serum, and may be related to the function of EphA3 in PCa progression.

Here, it was proposed that ADAM10 cleaves the EphA3/ephrin-A5 complex to promote PCa metastasis. A PCa cell line stably overexpressing ADAM10 and a PCa cell line with ADAM10 knockdown were constructed. The roles of ADAM10 in PCa metastasis and the mechanisms by which ADAM10 mediates PCa metastasis through EphA3 and ephrin-A5 were investigated in cultured ADAM10 intervened PCa cell lines, and these cell line xenograft mice. The potential of ephrin-A5 as a clinical adjunctive diagnostic marker was also assessed.

## Results

### ADAM10 expression in stable established PCa cell lines

**Fig. 1:**
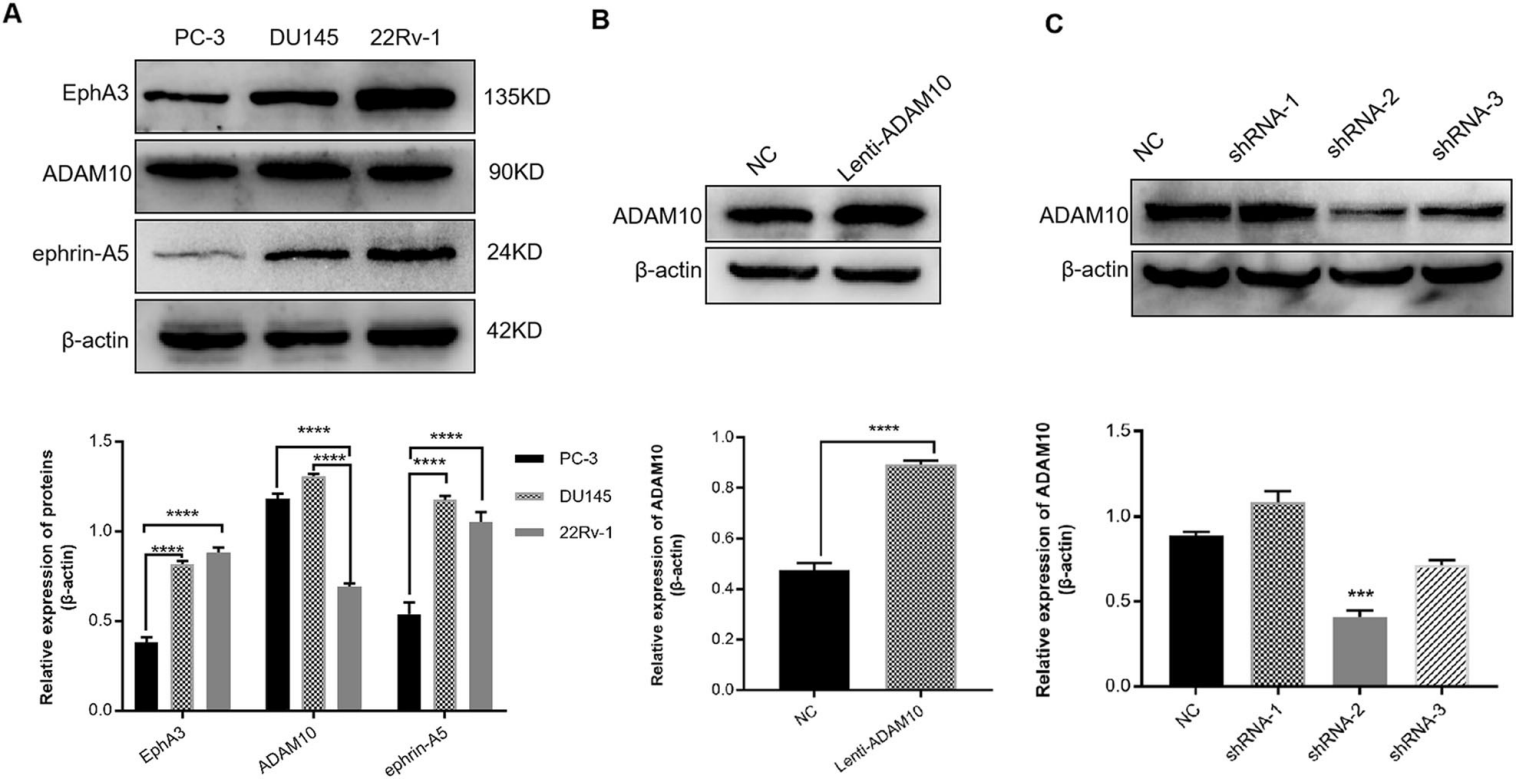
Measurement of ADAM10, EphA3 and ephrin-A5 proteins levels and construction of stable cell lines. **A** The expression of three proteins in PC-3, DU145, 22Rv-1 cell lines were detected by Western blotting (top), quantification of protein expression was normalized to β-actin(bottom). **B** Western blotting confirmed the overexpression of ADAM10 in DU145 cell line (top), quantification of protein expression was normalized to β-actin(bottom). **C** Western blotting confirmed the knockdown of ADAM10 in DU145 cell line (top), quantification of protein expression was normalized to β-actin(bottom). *P < 0.05, **P < 0.01, ***P < 0.001, ****P < 0.0001.

To investigate the function of ADAM10 in PCa progression, we firstly analyzed the endogenous expression of ADAM10, EphA3, and ephrin-A5 in three commonly used PCa cell lines (PC-3, DU145, 22Rv-1). ADAM10 protein existed in multiple cell lines, but EphA3 and ephrin-A5 protein were differentially expressed. A stronger expression of the three proteins were detected in 22Rv-1 cell line, while the expression of EphA3 and ephrin-A5 in DU145 was significantly higher than that in PC-3 (Fig. 1A). Therefore, DU145 cells were selected for the functional analysis of ADAM10. Expression of ADAM10 was significantly (*P* < 0.05) increased in the DU145 cells infected with lentivirus overexpressing ADAM10 as compared with the control group (Fig. 1B). ADAM10 expression was significantly (*P* < 0.05) inhibited in DU145 cells infected with shRNA-2 lentiviral particles (Fig. 1C). These results indicated that both ADAM10 overexpression DU145 cell line and ADAM10 knockdown DU145 cell lines were established.

### ADAM10 exerts pro-tumor effect in vitro

**Fig. 2:**
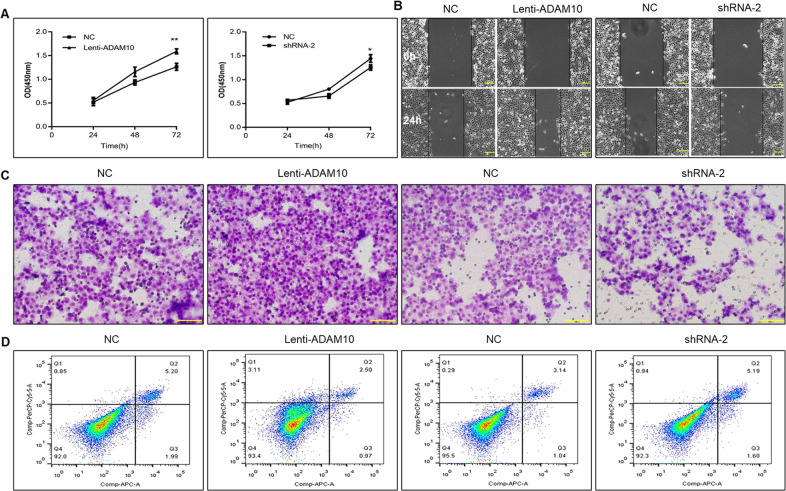
ADAM10 exerts pro-tumor effect in vitro. **A** Effect of ADAM10 on the proliferation of DU145 cells, Lenti-ADAM10 *vs* NC, **P < 0.01, shRNA-2 *vs* NC, *P < 0.05. **B** The effect of ADAM10 on the migration ability of DU145 cells was detected by scratch healing experiment, and the scratch healing rate was calculated, Lenti-ADAM10 *vs* NC, **P < 0.01, shRNA-2 *vs* NC, ***P < 0.001, Scale bar, 100 µm. **C** Trans-well assay was used to detect the effect of ADAM10 on the invasive ability of DU145 cells, and the number of lower chamber cells was counted and compared.Lenti-ADAM10 *vs* NC, ***P < 0.01, shRNA-2 *vs* NC, ***P < 0.001, Scale bar, 100 µm. **D** Effect of ADAM10 on apoptosis of DU145 cells was detected by flow cytometry, Lenti-ADAM10 *vs* NC, **P < 0.01, shRNA-2 *vs* NC, ***P < 0.001.

To investigate whether ADAM10 promoted PCa progression, the effect of ADAM10 on cell proliferation, apoptosis, migration, and invasion was evaluated in DU145 cells. Compared with control, ectopic expression of ADAM10 not only significantly increases the proliferation (Fig. 2A), migration (Fig. 2B), and invasion (Fig. 2C) but also inhibits apoptosis of DU145 cells (Fig. 2D). Meanwhile, the opposite effect was observed in ADAM10 depleted (Fig. 2) or GI254023X (a ADAM10 specific inhibitor, Supplementary Fig. S1) treated DU145 cells suggesting ADAM10 play a promoter role in PCa progression.

### Interaction of ADAM10, EphA3, ephrin-A5 in PCa cells

**Fig. 3:**
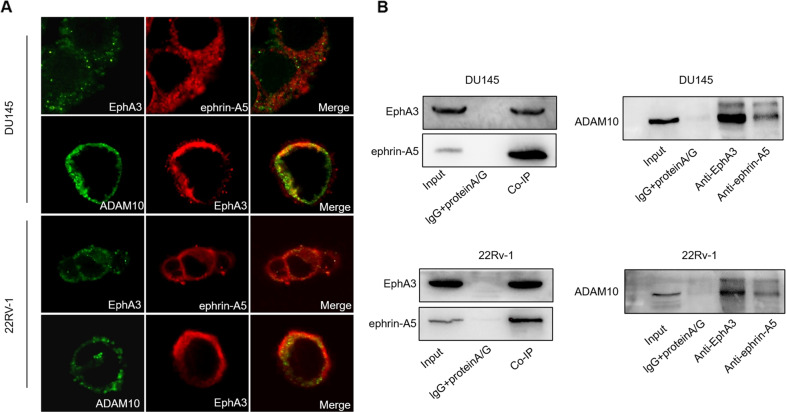
Interaction of ADAM10, EphA3, ephrin-A5 in cells. **A** Detection of cellular localization of ADAM10, EphA3, and ephrin-A5 by Immunofluorescence in DU145(top) and 22Rv-1(bottom). **B** The interaction of EphA3, ephrin-A5 and ADAM10 proteins was detected by co-immunoprecipitation in DU145(top) and 22Rv-1(bottom). Input: positive control, lgG+proteinA/G: negative control, Co-IP: using anti-EphA3 antibody or anti-ephrin-A5 antibody to detect immunoprecipitates.

Prior studies have confirmed that the pro-tumor role of ADAM10 is linked to the interaction among ADAM10, EphA3, and ephrin-A5. Thus, the distribution of ADAM10, EphA3 and ephrin-A5 in PCa cells was investigated. EphA3 and ephrin-A5 were mainly co-located on the cell membrane in DU145 and 22Rv-1 cells. And EphA3 strongly merged with ADAM10 in DU145 and 22Rv-1cell membrane (Fig. 3A). Co-immunoprecipitation indicated that ephrin-A5, EphA3 and ADAM10 physically contacted in DU145 and 22Rv-1 cells, suggesting that ephrin-A5, EphA3 and ADAM10 form complexes in PCa cells (Fig. 3B). These data implied that ephrin-A5 was a specific ligand of EphA3 in PCa cells, and ADAM10 also interacted with ephrin-A5 and EphA3 in vitro.

### ADAM10 induces the shedding and releasing of ephrin-A5 into the medium in vitro

**Fig. 4:**
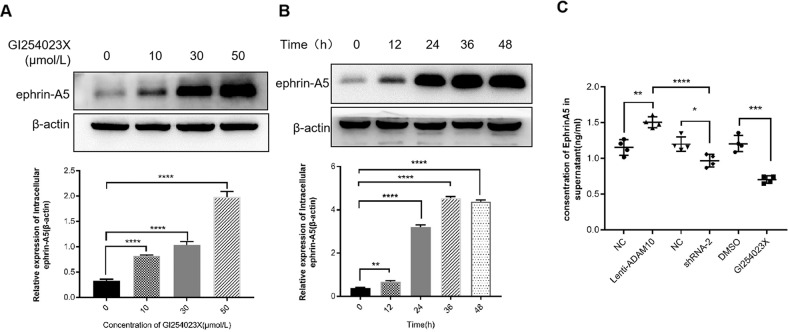
ADAM10 induces the shedding of ephrin-A5. **A** Detection of intracellular ephrin-A5 protein content by Western blotting. GI254023X solutions of different concentrations were treated for 24 h, and the protein content of ephrin-A5 in the cells was detected, ****P < 0.0001. **B** After being treated with 50 μmol/L GI254023X solution for different time, the content of ephrin-A5 protein in cells was detected. **P < 0.01, ****P < 0.0001. **C** Detection of ephrin-A5 content in cell culture medium by ELISA, *P < 0.05, **P < 0.01, ***P < 0.001, ****P < 0.0001.

As ephrin-A5 is a substrate of ADAM10 and plays a role in PCa progression, the relationship between GI254023X and the content of ephrin-A5 in PCa cells and cell culture medium was investigated. The intracellular protein level of ephrin-A5 increased gradually and reached a high level at 50μmol/L GI254023X treatment (Fig. 4A). So, 50μmol/L GI254023X was selected to treat PCa cells at different time points (12, 24, 36, and 48 h). It was showed that the intracellular level of ephrin-A5 protein gradually increased and reached a high level at 24 h (Fig. 4B). Therefore, treatment with 50 μmol/L GI254023X for 24 h was chosen for the subsequent experiments. Subsequently, we detected whether the ephrin-A5 content in cell culture medium has also changed accordingly. Compared to the untreated group, the concentration of ephrin-A5 was significantly(*P* < 0.05) increased in the culture medium of PCa cells with ADAM10 overexpression. In contrast, silencing of ADAM10 reduced ephrin-A5 secretion (Fig. 4C). The above experimental results indicated that ADAM10 could specifically cleave ephrin-A5 and release the soluble ephrin-A5 ectodomain into extracellular media.

### EphA3 fulfills pro-tumor function and ephrin-A5 reverses this effect in vitro

**Fig. 5:**
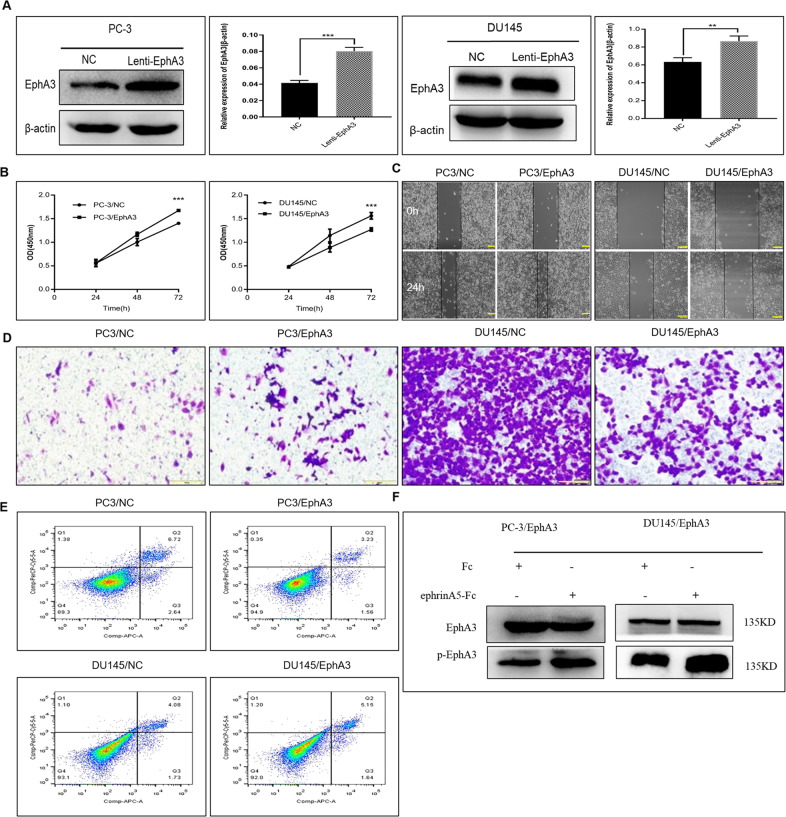
Overexpression of EphA3 exerts an opposite effect in DU145 and PC-3 cell lines. **A** The protein content of EphA3 in PC-3(left) and DU145(right) cells after lentivirus infection was detected by Western blotting, ***P < 0.001, **P < 0.01. **B** Effect of EphA3 on the proliferation of PC-3 and DU145 cells, PC-3/EphA3 *vs* PC-3/NC, ***P < 0.001, DU145/EphA3 *vs* DU145/NC, ***P < 0. 001. **C** The effect of EphA3 on the migration ability of PC-3 and DU145 cells was detected by scratch healing experiment. PC-3/EphA3 *vs* PC-3/NC, ***P < 0.001,DU145/EphA3 *vs* DU145/NC, ***P < 0. 001. Scale bar, 100 µm. **D** Trans-well assay was used to detect the effect of EphA3 on the invasive ability of PC-3 and DU145 cells, PC-3/EphA3 *vs* PC-3/NC, *P < 0.05,DU145/EphA3 *vs* DU145/NC, ***P < 0. 001. Scale bar, 100 µm. **E** Effect of EphA3 on apoptosis of PC-3 and DU145 cells was detected by flow cytometry, PC**-**3/EphA3 *vs* PC-3/NC, **P < 0.01,DU145/EphA3 *vs* DU145/NC, *P < 0. 05. **F** Effects of ephrinA5-Fc on phosphorylation of EphA3 protein.

Accumulated evidence indicates that the dual biological effects of EphA3 depend on whether it is stimulated by its ligand ephrin-A5. Thus, the biological functions of EphA3 were explored in PC-3 cells over-expressing EphA3 and low-expressing endogenous ephrin-A5 and DU145 cells over-expressing EphA3 and high-expressing endogenous ephrin-A5 (Fig. 5A). Ectopic expression of EphA3 in PC-3 cells with low expression of ephrin-A5 not only significantly inhibited PC-3 cell apoptosis but also obviously promoted PC-3 cell proliferation, migration and invasion compared to the control group, implying that EphA3 promotes PCa progression. Paradoxically, an opposite role was observed in the DU145 cells over-expressing EphA3 and high-expressing endogenous ephrin-A5 (Fig. 5B–E), suggesting that the expression of ephrin-A5 might be involved. Exogenous ephrinA5-Fc stimulation PC-3 cells with EphA3 over-expression, caused a significant reduction in cell growth, migration, and invasion, and an increased in cell apoptosis (Supplementary Fig. S2A–D). Meanwhile, it is worth noting that the phosphorylation level of EphA3 was significantly up-regulated by the stimulation of exogenous ephrin-A5 ligand in both stable DU145 cell lines (Fig. 5F), suggesting that ephrin-A5 can not only promote the phosphorylation of EphA3 but also make EphA3 play an anti-tumor effect in vitro. These results indicated that the dual function of EphA3 as tumor promoter and suppressor were regulated by the ephrin-A5 ligand.

### ADAM10 cleaves ephrin-A5 to promote PCa metastasis in vivo

**Fig. 6:**
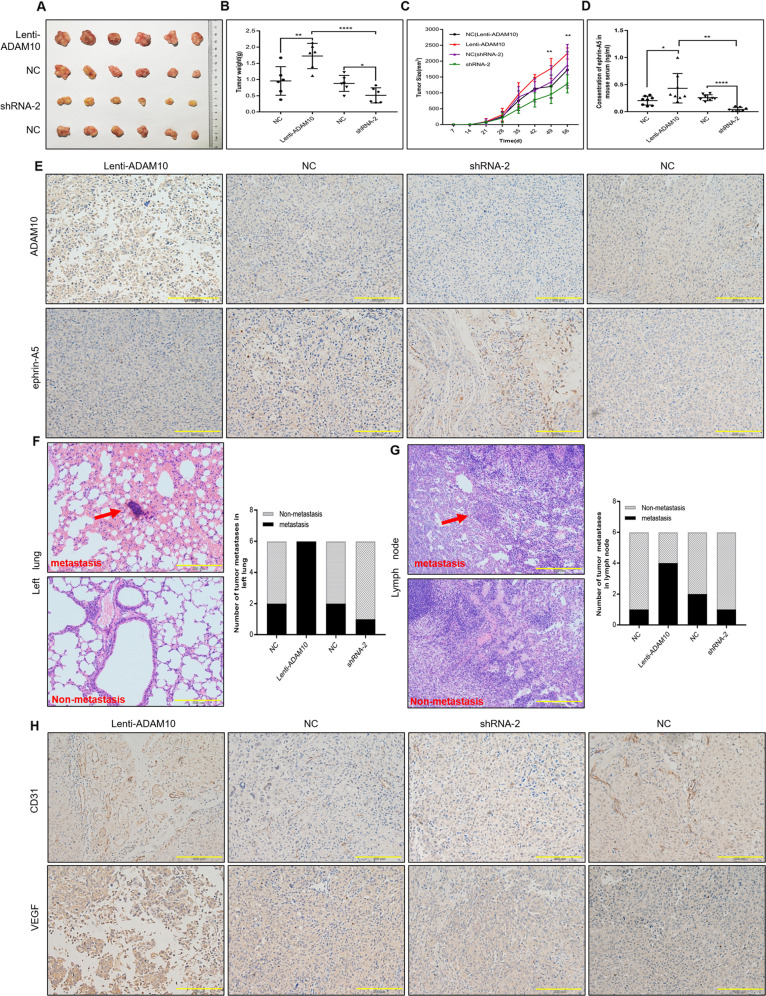
ADAM10 cleaves ephrin-A5 and promotes tumor metastasis in vivo. **A**–**C** Tumor volume and weight at the end of the experiment. The data are shown as the means ± sd (*n* = 6), *P < 0.05, **P < 0.01, ****P < 0.0001. **D** The serum ephrin-A5 content of nude mice in each treatment group was detected by ELISA (*n* = 6), *P < 0.05, **P < 0.01, ****P < 0.0001. **E** The expression levels of ADAM10 and ephrin-A5 in tumor tissues were detected by immunohistochemistry, Scale bar, 200 µm. **F** HE staining was used to detect tumor metastasis in the left lung of nude mice (left), statistics of tumor metastasis rate in each treatment group (right). Scale bar, 200 µm. **G** HE staining was used to detect tumor metastasis in the lymph node of nude mice (left), statistics of tumor metastasis rate in each treatment group (right). Scale bar, 200 µm. **H** The expression levels of CD31 and VEGF in tumor tissues were detected by immunohistochemistry. Scale bar, 200 µm.

To further validate whether ADAM10 could reduce EphA3 phosphorylation by cleaving ephrin-A5 to promote tumor progression in vivo, subcutaneous xenograft models were established using DU145-ADAM10, DU145-shRNA2, and their control cells in nude mice. Consistent with the proliferation of DU145 cell in vitro, the primary tumors of DU145-ADAM10 grew faster than the corresponding DU145/Control tumors. Similarly, the average tumor volume and weight of the DU145-ADAM10 primary tumors were also significantly much than those of the corresponding DU145/Control tumors. Whereas the opposite results were observed in the primary tumors of DU145-shRNA2, suggesting that ADAM10 can promote tumor growth in vivo (Fig. 6A–C). Subsequently, we detected the ephrin-A5 content in the serum of nude mice and found that the overexpression of ADAM10 could significantly increase the level of serum ephrin-A5, while it was significantly decreased after the elimination of ADAM10 (Fig. 6D), suggesting that serum ephrin-A5 may be a potential PCa marker. Contrary to the content of serum ephrin-A5, the ephrin-A5 expression was significantly reduced in DU145-ADAM10 primary tumors, which was almost undetectable. And, the opposite result was also observed in the primary tumor tissue of DU145-shRNA2 (Fig. 6E). These data supported that ADAM10 promoted the release of ephrin-A5 to the serum and PCa growth in vivo.

Next, HE staining was performed to detect whether ADAM10 could promote tumor metastasis in the left lung and lymph nodes of the nude mice. Tumor metastatic foci were observed in all the left lung tissues of the nude mice in the ADAM10 overexpression group, while only one of the six nude mice with ADAM10 knockdown had metastasis in the left lung tissues (Fig. 6F). Similarly, tumor metastatic foci were also existed in four out of six nude mice in ADAM10 overexpressing group, but only one nude mouse had metastasis in lymph node tissue after ADAM10 being knocked down (Fig. 6G). The expression levels of CD31 and VEGF, the markers of angiogenesis, were significantly increased in the tumor tissues of the ADAM10 overexpression group, suggesting that ADAM10 promote tumor metastasis through enhancing angiogenesis (Fig. 6H)

### Ephrin-A5 could be a biomarker for PCa metastasis

**Fig. 7:**
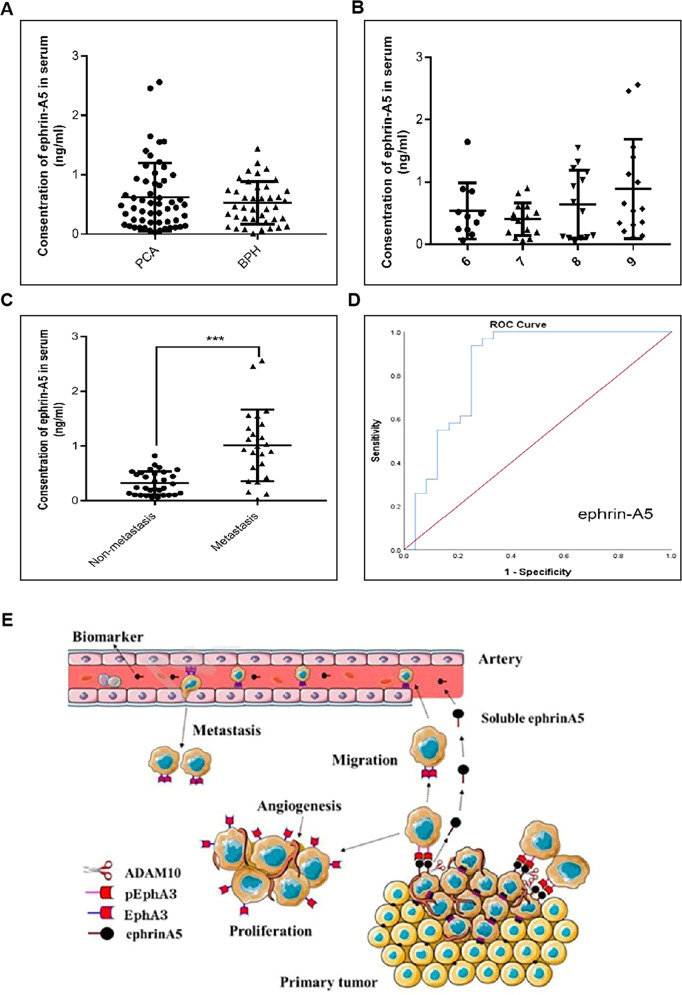
Detection of serum ephrin-A5 by ELISA. **A** Serum ephrin-A5 levels were measured by ELISA in patients with prostate cancer (*n* = 55) and benign prostatic hyperplasia (*n* = 40), PCA *vs* BPH, *P* > 0.05. **B** The content of ephrin-A5 in serum of patients with different Gleason score. **C** Serum ephrin-A5 levels were detected by ELISA in patients with metastatic prostate cancer (*n* = 24) and non-metastatic prostate cancer (*n* = 31), Non-metastasis *vs* Metastasis, ***P < 0.001. **D** Evaluation of Serum ephrin-A5 as a Metastasis Marker of Prostate Cancer by ROC. **E** A schematic representation of prostate cancer metastasis caused by ADAM10 and EphA3/ephrin-A5 complex.

To further investigate the clinical application value of serum ephrin-A5 as a PCa marker, we assessed the content of serum ephrin-A5 in 55 patients with PCa and 40 patients with BPH and found that the serum concentration of ephrin-A5 in the PCa group was not significantly different from that in the BPH group (Fig. 7A). And, no correlation was observed between serum ephrin-A5 level and patient's Gleason score (Fig. 7B). However, the average of serum ephrin-A5 concentrations was 3.4 fold higher in metastatic PCa patients (1.01 ± 0.13 ng/ml) compared to non-metastatic PCa patients (0.32 ± 0.038 ng/ml) (Fig. 7C). The AUC of serum ephrin-A5 in the identification of PCa metastasis is 0.843 (95%, CI: 0.723, 0.962), with a sensitivity of 93.5% and a specificity of 75%. These results suggest that serum ephrin-A5 has the potential to be used as a diagnostic marker for metastatic PCa (Fig. 7D).

## Discussion

In the present study, PCa cell lines with intervened ADAM10 expression were established. it was found that intervention of ADAM10 expression influenced PCa metastasis in cultured cells and PCa cell xenograft mice, and angiogenesis in PCa cell xenograft mice. Intervention of ADAM10 expression affected both intracellular and intercellular levels of ephrin-A5 in vitro and in vivo. ADAM10, EphA3 and ephrin-A5 formed complex and co-localized on the cell membrane. Overexpression of EphA3 in PC-3 cells with low expression of ephrin-A5 promoted PCa progression while over-expressing EphA3 in the DU145 cells with high-expressing endogenous ephrin-A5 inhibited PCa progression. Exogenous ephrinA5-Fc stimulation inhibited PCa progression in PC-3 cells with EphA3 over-expression, and up-regulated the phosphorylation level of EphA3 in both stable DU145 cell lines. The serum ephrin-A5 concentration was higher in metastatic PCa patients than non-metastatic PCa patients. These results demonstrated that ADAM10 promoted PCa metastasis through interacting with EphA3/ephrin-A5 complex and facilitating the release of ephrin-A5 out of PCa cells. The human serum concentration of ephrin-A5 may be used as a diagnostic marker for metastatic PCa.

Accumulated evidence has demonstrated that ADAM10 participates in the occurrence and development of various tumors, such as hepatocellular carcinoma [9], hypopharyngeal cancer [10], esophageal cancer [11], and PCa [13]. The high levels of ADAM10 protein in the three PCa cell lines agree with the observation by Wetzel et al. [13]. Overexpression of ADAM10 promoted the proliferation, migration and invasion and inhibited apoptosis in cultured PCa cells, whereas knocking down ADAM10 or administration of ADAM10 inhibitor GI254023X led to an opposite effect. These results indicated that ADAM10 might promote PCa metastasis, which was confirmed by our observations that overexpression of ADAM10 in PCa cell xenograft mice facilitated tumor growth and increased the tumor metastatic foci in the left lung and lymph nodes of the nude mice and supported by the observation that ADAM10 promoted PCa metastasis. The ADAM10 augmented expression levels of angiogenesis markers CD31 and VEGF in tumor tissues suggest that ADAM10 promote angiogenesis.

It was found that ADAM10 bound EphA3 and ephrin-A5 to form complex and these proteins co-localized on the cell membrane, indicating that ADAM10 physically interacts with EphA3/ephrin-A5 complex in PCa cells [16, 19, 24]. EphA3, an essential regulatory target of ADAM10 through cleaving its primary ligand ephrin-A5, is a member of the receptor tyrosine kinase family, and has been reported to have dual biological effects in tumor progression [16]. Up-regulation of EphA3 in PCa is associated with accelerated cell migration and invasion, increased angiogenesis, and a poor prognosis [17]. Conversely, EphA3 suppressed tumors by disrupting the tumor stromal microenvironment in mouse xenografts [18]. These opposing roles may be regulated by its primary ligand ephrin-A5, which induces the phosphorylation of EphA3 receptor and triggers the downstream signal transduction involved in tumor suppression [25, 26]. In our study, overexpression of EphA3 in PC-3 cells with low expression of ephrin-A5 promoted PCa progression while over-expressing EphA3 in the DU145 cells with high expression of endogenous ephrin-A5 inhibited PCa progression. Exogenous ephrinA5-Fc stimulation inhibited PCa progression in PC-3 cells with EphA3 over-expression, and up-regulated the phosphorylation level of EphA3 in both stable DU145 cell lines. All these results indicated that overexpression of EphA3 in PC-3 cells with low level of endogenous ephrin-A5 protein led to a kinase-independent cancer-promoting effect, while overexpression of EphA3 in DU145 cells with high level of endogenous ephrin-A5 protein caused a kinase-dependent inhibitory effect. ADAM10 could mediate the hydrolysis of ephrin-A5 and its release into the cell culture medium in vitro, which was in agree with the previous study [30]. In DU145 cells xenograft mice, overexpression of ADAM10 increased the level of serum ephrin-A5 and decreased the content of ephrin-A5 inside DU145-ADAM10 primary tumors, while knock-down of ADAM10 led to an opposite effect. Taken together, all the results support that ADAM10 physically interacts with EphA3/ephrin-A5 complex, triggers dissociation of ephrin-A5 from EphA3 and the release of ephrin-A5 out of the PCa cells, relieves the inhibitory effect of ephrin-A5 on EphA3 and promotes metastasis in PCa.

Currently, since prostate-specific antigen (PSA) screening is not widely available, prostate cancer in many patients has already been metastatic at the initial diagnosis. Though some prostate cancer metastasis-related markers, such as serum miR-141 and exosomes-derived miR-141-3p has been found [31, 32], it is far from clinical application. Our finding that ephrin-A5 is disassociated from EphA3 and released outside the cell by ADAM10 suggests that ephrin-A5 may have certain diagnostic value in prostate cancer metastasis [33]. The similar ephrin-A5 content in serum of the patients with prostate cancer and BPH indicates that serum ephrin-A5 cannot be used as a marker of common prostate malignancy. A trend in the increase of serum ephrin-A5 content related to Gleason score (data not shown) and a higher serum ephrin-A5 content in metastatic PCa patients than non-metastatic PCa patients imply that serum ephrin-A5 protein level could be a potent marker for PCa metastasis. ROC curves analysis shown that serum ephrin-A5 could distinguish patients with metastatic PCa and non-metastatic with high sensitivity and specificity. The above results indicate that serum ephrin-A5 content is a new marker for prostate cancer metastasis.

In addition, it is noteworthy that ADAM10 is a promising therapeutic target for PCa. Since ADAM10 can enhance the anti-apoptotic ability of tumor cells by hydrolyzing Fas ligand. Targeted inhibition of ADAM10 activity is a potential therapeutic approach for apoptosis-resistant prostate cancer [34]. Inhibition of ADAM10 expression can also inhibit Notch1 signal transduction and make drug-resistant cells re-sensitive to enzalamide [35]. A monoclonal antibody against the Eph/Ephrin recognition site of ADAM10 inhibits protease cleavage with fewer toxic and side effects [36]. In this study, the ADAM10 specific inhibitor GI254023X suppressed PCa progression at the cellular level. It would be exhilarating that if an inhibitor of ADAM10 could be developed and applied in the clinic to decrease the mortality by reducing the metastasis of prostate cancer, which is of great significance to patients and society.

In conclusion, ADAM10, EphA3 and ephrin-A5 physically contacted to form a complex, and co-localized on cell membrane in PCa cells. ADAM10-cleaved ephrin-A5, disassociated ephrin-A5 from EphA3, released ephrin-A5 outside of cells, relieved the inhibition of ephrin-A5 on EphA3, and promoted metastasis by increasing angiogenesis in PCa. Serum ephrin-A5 can be used as a new biomarker for PCa metastasis.

## Materials and methods

### Sample information

From January 2018 to July 2019, serum samples from 55 patients with PCa and 40 patients with benign prostate hyperplasia (BPH) were collected and stored in a refrigerator at −80 °C for later use. The study was approved by the Ethics Committee of the affiliated Hospital of Xuzhou Medical University and informed consent was signed by patients or their families. All cases were diagnosed by the department of clinical pathology, Hospital of Xuzhou Medical University. The basic clinical data of the patients are shown in Supplementary Table S1.

### Cell culture

PC-3, DU145, 22Rv-1 cell lines were purchased from the Cell Bank of Chinese Academy of Sciences. The three types of PCa cells were cultured in 1640 medium supplemented with 10% fetal bovine serum (Clark, Australia), at 37 °C in a humidity cell incubator containing 5% CO_2_.

### Establishment of stable cell lines

In this project, ADAM10 and EphA3 gene were stably overexpressed or knocked down in PCa cell lines. The full-length cDNA of human ADAM10 or EphA3 was cloned into lentivirus vectors at certain site. Lentiviruses expressing ADAM10 shRNA-1 (ATGGTTATCTTACGACTGTTA), ADAM10 shRNA-2 (GACCACCCACGAATGGTTATC), and shRNA-3: GGCGTATTGGAGACGAGTTTA) or control shRNA (AGGUAGUGUAAUCGCCUUGTT) were used for RNA interference. Recombinant plasmid and the auxiliary plasmid psPAX, pMD2.G were introduced into 293 T cells in the presence of PEI reagent (Proteintech, Chicago, USA) to generate lentiviruses. Subsequently, lentivirus was used to infection PCa cells when the cell fusion density was 30 to 40%. One week after transfection, puromycin (Vicmed, Xuzhou, China) was used to screen the positive cells (PC-3: 2 μg/ml, DU145:4 μg ml), and the positive cells were selected by detecting the expression of protein by Western blotting.

### Cell proliferation

A total of 5000 PCa cells were spread in 96-well plates with 5 replicates in each group. At 24, 48, and 72 h after culture, 10 µl CCK-8 reagent (Keygen Biotech, Nanjing, China) was added to each well and incubated at 37 °C for 2.5 h. The absorbance was read at 450 nm with an automatic microplate reader for the generation of a growth curve.

### Wound healing assay

1 × 10^5^ PCa cells were laid in a 6-well plate. When cells reached confluency, scratches were made using pipette tips. Subsequently, serum-free 1640 culture medium was replaced, and pictures were taken at the same position at 0 h and 24 h, respectively. The experiment was repeated for three times, and Image J software was used to detect the scratch healing area and calculate the healing rate.

### Invasion assay

The Matrigel (Corning Costar, New York, USA) diluted with serum-free medium was pre-laid on the upper layer of the chamber to simulate the extracellular environment. A total of 5000 PCa cells suspended in 100 μl serum-free medium were added to the upper chamber, and the lower chamber contained 10% FBS serves as a chemoattractant. After 48 h of culture in a 37 °C incubator, the chamber was taken out, fixed with 4% paraformaldehyde, and stained with 0.1% crystal violet. After wiping off the superior ventricular cells, five random visual fields were photographed under the microscope, and the number of cells was counted. The experiment was repeated three times, and the average value was taken.

### Apoptosis experiment

2 × 10^5^ cells in logarithmic growth stage were collected and resuspended by 200 μl binding buffer. Then the cells were transferred into the flow tube, and blank group, single standard group, control group, and experimental group were set up. Blank group: cells without any reagent; Single bid group (Annexin V-APC): Annexin V-APC 5 µl was added. Single bid group (Annexin 7-AAD): 5 µl Annexin 7-AAD was added; Annexin 7-AAD 5 µl and Annexin V-APC 5 µl were added to the control group and the experimental group, respectively. At room temperature, the foil was wrapped in dark and incubated for 15 min. Flow cytometry was used for detection within 1 h, and the experiment was repeated for three times.

### Detection of soluble ephrin-A5

About 1 × 10^6^ PCa cells were cultured overnight in one well of a 6-well plate. At the next day, the plates were washed with PBS for twice, and then 2 ml serum-free 1640 medium was added. After continuous culture for 24 h, the cell culture medium was collected in a tube and centrifuged for 5 min at 2000 × *g*, then the supernatant was obtained. Then, the supernatant was frozen in a refrigerator at −80 °C and then put in a freeze-dryer overnight. The freeze-dried powder was collected and dissolved in 200 μl sample diluent from Human ephrin-A5 ELISA Kit (Fine Biotech, Wuhan, China) and detected according to the instructions. In addition, the kit was used to detect ephrin-A5 in serum samples.

### Immunofluorescence

The cell immunofluorescence staining was performed following the procedure reported by another research group [37]. Primary antibodies against ADAM10 (CST, Boston, USA), EphA3 (Santa Cruz, California, USA), ephrin-A5 (R&D systems, Minnesota, USA) were applied at a ratio of 1:200. Fluorescent secondary antibodies (Alexa488, Alexa594, Thermo Scientific, Massachusetts, USA) were also applied at a ratio of 1:200. The images were observed and collected using a laser confocal microscope.

### Co-immunoprecipitation

Cells were divided into the positive control group, negative control group and experimental group; and the protein lysate of PCa cells was extracted according to the literature [38], 1.5 μg antibody [ADAM10 (CST, USA), EphA3 (Santa Cruz, USA), ephrin-A5 (R&D systems, USA), or ephrin-A5 (Santa Cruz, USA) was added to the test group and 1.5 μg homologous lg monoclonal antibody was added to the negative control group. After incubated in a shaker at 4 °C for 12 h, the target protein was enriched by 20 μl washed protein A/G agarose magnetic beads(MCE, USA). The precipitated proteins were detected using Western blotting.

### Detection of EphA3 phosphorylation

EphrinA5-Fc and Fc were purchased from R&D systems, USA. Anti-EphA3 antibody and protein A/G magnetic beads were used to enrich EphA3 protein in cell protein lysate. The EphA3 enriched cell lysate was added with 1/5 volume of 5 × SDS sample buffer in a metal bath at 100 °C for 10 min. Phosphorylated EphA3 was detected by Western blotting using the primary antibody (against universal phosphorylated tyrosine (1:1000, Abcam, UK).

### Subcutaneous xenograft tumor model

Subcutaneous tumorigenesis was performed in nude mice according to our previous study [39]. A total of 24 four-week-old male BALB/C mice were purchased from Jiangsu Jicui Yaokang Biotechnology and reared under specific pathogen-free conditions(SPF). The tumorigenic tumor cells were divided into four groups: DU145-ADAM10, DU145-shRNA2 and their control groups. 1 × 10^7^ cells were suspended in 100 μl saline and mixed with 100 μl Matrigel (Corning Costar, New York, USA), and then injected under the right axilla of nude mice (6 mice/group, randomly).

The state of nude mice was observed daily, and the tumor was measured using a vernier caliper every week, the tumor volume was calculated using the following calculation formula: volume = width ^2^ × length × 0.52. On 56th day of rearing, they were sacrificed after anesthesia. The blood was taken after decapitation, and the primary tumor tissue was stripped and weighed. At the same time, the right lung and the right lymph node tissue of the nude mice were taken and fixed in 4% formalin solution.This study was approved by the Animal Care Ethics Committee of Xuzhou Medical University (LA20210226419).

### Immunohistochemistry

The immunohistochemical staining was conducted according to the literature [40]. The primary antibodies against CD31 (Servicbeio, China), VEGF (Servicbeio, China), ADAM10 (Servicbeio, China), and ephrin-A5 (Santa Cruz, China) were applied at a ratio of 1:2000. HRP labeled goats against rabbits’ secondary antibody (Servicbeio, China) and HRP labeled goats against mice secondary antibody (Servicbeio, China) were applied at a ratio of 1:200.

## Statistics

SPSS 21.0 software and GraphPad Prism 7.0 software were used for all the experimental data analysis. The difference between two independent samples was detected by t-test. One-way ANOVA was performed to compare groups with multiple samples. *P* < 0.05 was considered statistically significant.

## Supplementary information

GI254023X treatment showed anticancer effect in DU145 cells

ephrinA5-Fc treatment reversed the oncogenic effect of EphA3 overexpression on PC-3 cell lines

Clinicopathological data of patients with prostate cancer and prostate hyperplasia

Reproducibility checklist

## Data Availability

The datasets generated and/or analyzed during the current study are available from the corresponding author on reasonable request.
