# Supplementary material for: ADAM10-cleaved ephrin-A5 contributes to prostate cancer metastasis
Source: Cell Death Dis. 2022 May 12;13(5):453. doi: 10.1038/s41419-022-04893-8 (PMC9098485; doi:10.1038/s41419-022-04893-8)
Supplement: Supplementary file 1 — GI254023X treatment showed anticancer effect in DU145 cells [file 41419_2022_4893_MOESM1_ESM.docx]

**
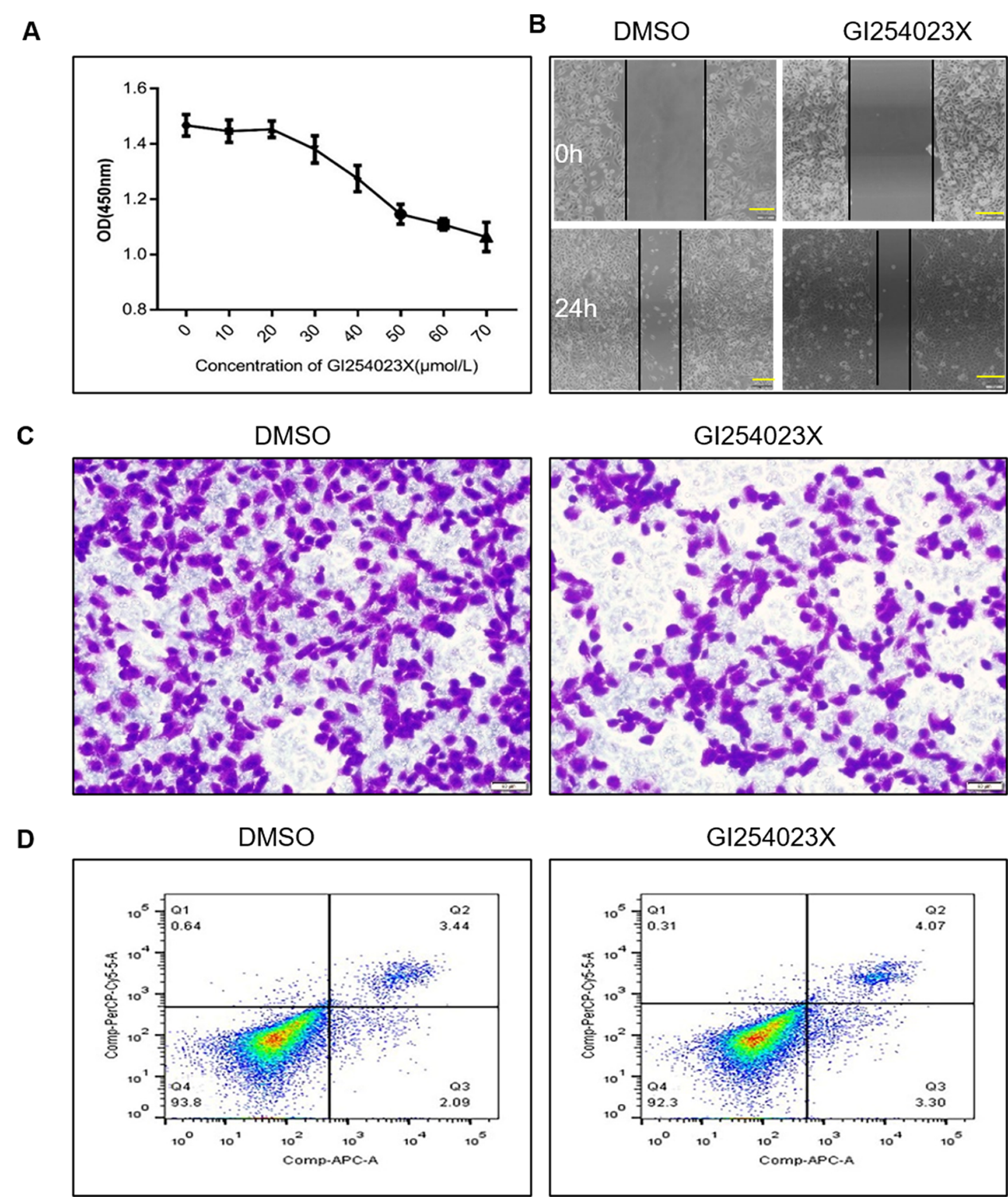
**

**Supplementary Fig. S1 GI254023X treatment showed anticancer effect in DU145 cells. A.** DU145 cell proliferation after treatment with different concentrations of GI254023X. **B.** The effect of GI254023X on the migration ability of DU145 cells was detected by scratch healing experiment, and the scratch healing rate was calculated, DMSO *vs* GI254023X, ***P*<0.01, Scale bar, 100 µm. **C.** Trans-well assay was used to detect the effect of GI254023X on the invasive ability of DU145 cells, and the number of lower chamber cells was counted and compared. DMSO vs GI254023X, ****P*<0.01, Scale bar, 100 µm. **D.** Effect of GI254023X on apoptosis of DU145 cells detected by flow cytometry, DMSO vs GI254023X, ***P<*0.01.
