# Supplementary material for: ADAM10-cleaved ephrin-A5 contributes to prostate cancer metastasis
Source: Cell Death Dis. 2022 May 12;13(5):453. doi: 10.1038/s41419-022-04893-8 (PMC9098485; doi:10.1038/s41419-022-04893-8)
Supplement: Supplementary file 2 — ephrinA5-Fc treatment reversed the oncogenic effect of EphA3 overexpression on PC-3 cell lines [file 41419_2022_4893_MOESM2_ESM.docx]

**
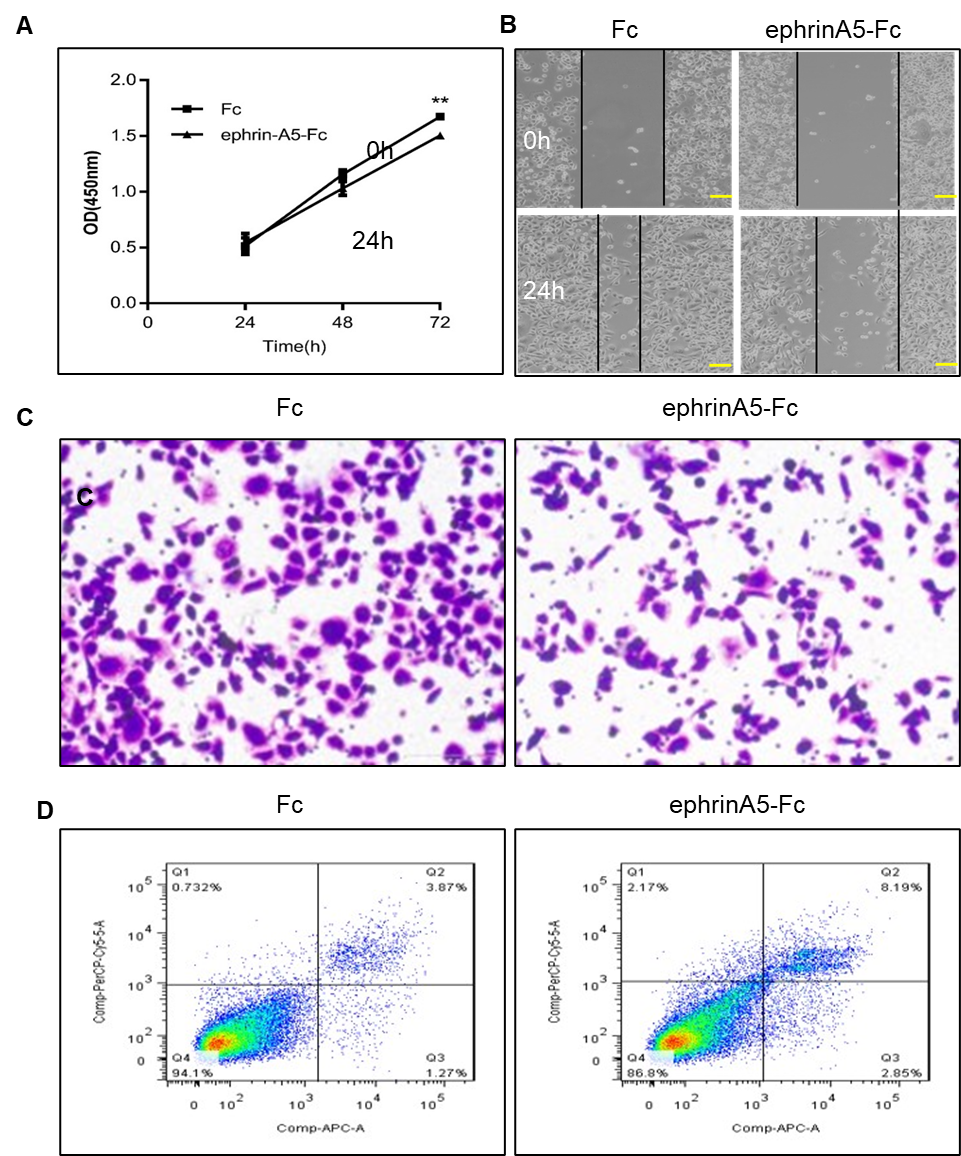
**

**Supplementary Fig.S2** **ephrinA5-Fc treatment reversed the oncogenic effect of EphA3 overexpression on PC-3 cell lines.** **A**. PC-3 cell proliferation after treatment with Fc or ephrinA5-Fc. Cells were plated in 96-well plates and then serum-deprived for 24 hours. After stimulation of 1.0μg/ml of ephrinA5-Fc or Fc for 15 minutes, cell viability from 24 hours to 72 hours in culture was determined, Fc vs ephrinA5-Fc, ***P*<0.01. **B**. The migration ability of PC-3 cells after treatment with Fc or ephrinA5-Fc. Cells were serum-deprived for 24hours, scratched with a tip, and photographed (T0). After stimulation of 1.0μg/ml of ephrinA5-Fc or Fc for 15 minutes, cells were cultured in complete medium for 24 hours and photographed (T24), Fc vs ephrinA5-Fc, ***P*<0.01, Scale bar, 100 µm. C. The invasive ability of PC-3 cells after treatment with Fc or ephrinA5-Fc. A total of 5×10^3^ cells were plated in trans-well inserts, treated with 1.0μg/ml of ephrin-A5 ligand or Fc for 15 minutes and cultured for 24 hours, Fc vs ephrinA5-Fc, ***P*<0.01, Scale bar, 100 µm. D. PC-3 cell apoptosis after treatment with Fc or ephrinA5-Fc. A total of 5×10^6^ PC-3 cells overexpressing EphA3 receptor were serum-deprived for 24 hours, and treated with 1.0μg/ml of ephrin-A5 ligand or Fc for 15 minutes. Then, cells were stained using Annexin V-APC and quantified by FACS, Fc vs ephrinA5-Fc, **P*<0.05.
