# Supplementary material for: ADAM10-cleaved ephrin-A5 contributes to prostate cancer metastasis
Source: Cell Death Dis. 2022 May 12;13(5):453. doi: 10.1038/s41419-022-04893-8 (PMC9098485; doi:10.1038/s41419-022-04893-8)
Supplement: Supplementary file 3 — Clinicopathological data of patients with prostate cancer and prostate hyperplasia [file 41419_2022_4893_MOESM3_ESM.docx]

**Supplementary Table S1: Clinicopathological data of patients with prostate cancer and prostate hyperplasia**

|  | PCA | BPH |
| --- | --- | --- |
| Case, n | 55 | 40 |
| Age (mean ± SD, years) | 72.22 ± 1.133 | 70.53 ± 1.265 |
| Metastasis |  |  |
| Negative | 24 |  |
| Positive | 31 |  |
| Gleason Score |  |  |
| 6 | 11 |  |
| 7 | 15 |  |
| 8 | 14 |  |
| 9 | 15 |  |
| PSA (ng/ml) | 109.4 ± 17.04 | 16.2 ± 2.731 |
